# Supplementary material for: Temporal convolutional networks predict dynamic oxygen uptake response from wearable sensors across exercise intensities
Source: NPJ Digit Med. 2021 Nov 11;4:156. doi: 10.1038/s41746-021-00531-3 (PMC8586225; doi:10.1038/s41746-021-00531-3)
Supplement: Supplementary file 1 — Supplementary Information [file 41746_2021_531_MOESM1_ESM.pdf]

Supplementary Information

Optimization of Number of Trees in Random Forest Model

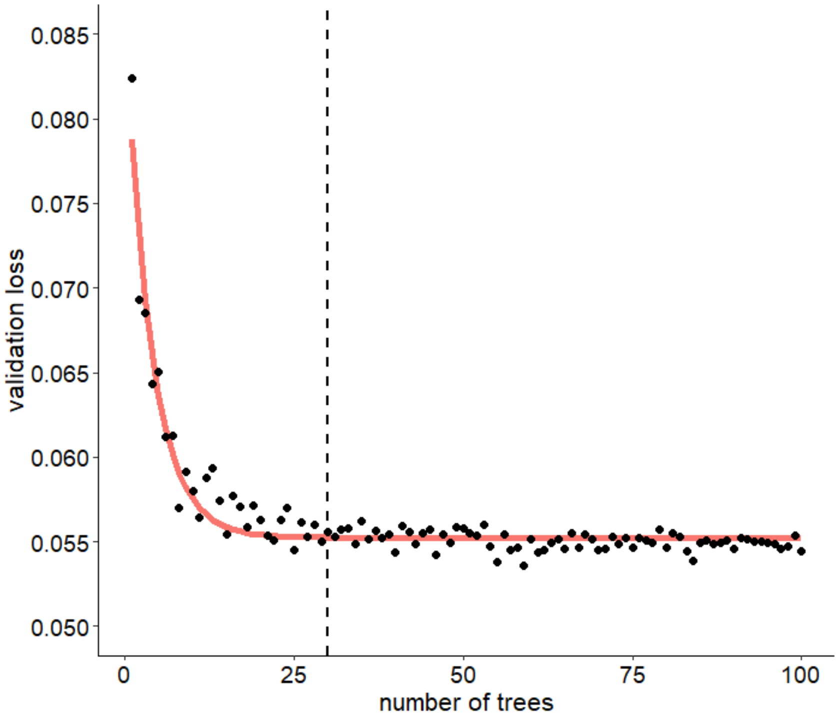

Supplementary Figure 1: Random forest optimal tree selection

Independent random forest models were trained with an increasing number of trees (1 to 100). Validation loss (mean squared error) was used to identify a sufficient number of trees to include in the model while minimizing both the model error and model size. Thirty trees were selected as the optimal model size, as the validation loss appeared to plateau at this point (dashed line). An exponential fit of the data (red line) is superimposed for enhanced visualization.

All TCN-VO2 Models Ordered By Validation Loss

This table presents the validation results of the hyperparameter values and receptive field ordered by validation loss (ascending). Generally, we see the performance decrease as the receptive field gets smaller. Only 16 and 24 filters were shown here for visual clarity.

Supplementary Table 1: All TCN-VO2 models ordered by validation loss

Number of filters	Kernel size (s)	Max dilation	Receptive field (s)	Validation loss
24	8	16	218	0.02506
16	7	16	187	0.02525
24	7	8	91	0.02546
24	6	8	76	0.02556
16	8	16	218	0.02558
16	6	8	76	0.02585
16	7	8	91	0.02586
24	8	8	106	0.02632
24	6	16	156	0.02649
24	4	16	94	0.02659
16	8	8	106	0.02665
16	4	16	94	0.02683
24	5	8	61	0.02690
24	5	16	125	0.02720
24	3	16	63	0.02754
16	6	16	156	0.02784
16	5	16	125	0.02795
24	7	16	187	0.02859
24	4	8	46	0.02889
24	8	4	50	0.02942
16	4	8	46	0.02954
16	3	16	63	0.02956
16	8	4	50	0.02994
16	5	8	61	0.03030
24	7	4	43	0.03177
24	6	4	36	0.03180
16	7	4	43	0.03241
16	6	4	36	0.03396
16	2	16	32	0.03400
16	3	8	31	0.03404
24	2	16	32	0.03451
24	3	8	31	0.03477
16	5	4	29	0.03514
24	8	2	22	0.03666
24	5	4	29	0.03703
16	7	2	19	0.03773
16	8	2	22	0.03797
16	4	4	22	0.03813
24	4	4	22	0.03945
24	7	2	19	0.04057
16	2	8	16	0.04136
24	2	8	16	0.04201
16	6	2	16	0.04205
24	1	8	1	0.04211
24	6	2	16	0.04238
24	5	2	13	0.04266
16	7	1	7	0.04309
24	3	2	7	0.04326
16	3	4	15	0.04330
24	8	1	8	0.04359
24	4	2	10	0.04366
16	5	2	13	0.04377
16	4	2	10	0.04380
24	7	1	7	0.04388
24	3	4	15	0.04407
16	1	8	1	0.04423
24	6	1	6	0.04435
16	8	1	8	0.04442
16	2	2	4	0.04458
24	2	2	4	0.04506
24	2	1	2	0.04516
24	1	16	1	0.04518
16	3	2	7	0.04525
16	6	1	6	0.04545
24	3	1	3	0.04546
24	1	2	1	0.04565
16	3	1	3	0.04593
16	4	1	4	0.04612
16	5	1	5	0.04628
16	1	4	1	0.04650
24	2	4	8	0.04668
24	1	4	1	0.04690
24	5	1	5	0.04692
16	2	1	2	0.04713
16	1	1	1	0.04811
16	2	4	8	0.04851
24	4	1	4	0.04880
16	1	16	1	0.04889
24	1	1	1	0.04895
16	1	2	1	0.05497
