# Supplementary material for: Temporal convolutional networks predict dynamic oxygen uptake response from wearable sensors across exercise intensities
Source: NPJ Digit Med. 2021 Nov 11;4:156. doi: 10.1038/s41746-021-00531-3 (PMC8586225; doi:10.1038/s41746-021-00531-3)
Supplement: Supplementary file 2 — Reporting Summary [file 41746_2021_531_MOESM2_ESM.pdf]

nature research

Corresponding author(s): Robert Amelard

Last updated by author(s): Aug 17, 2021

Reporting Summary

Nature Research wishes to improve the reproducibility of the work that we publish. This form provides structure for consistency and transparency in reporting. For further information on Nature Research policies, see our Editorial Policies and the Editorial Policy Checklist.

Statistics

For all statistical analyses, confirm that the following items are present in the figure legend, table legend, main text, or Methods section.

n/a	Confirmed	
☐	☒	The exact sample size (*n*) for each experimental group/condition, given as a discrete number and unit of measurement
☐	☒	A statement on whether measurements were taken from distinct samples or whether the same sample was measured repeatedly
☐	☒	The statistical test(s) used AND whether they are one- or two-sided<br>*Only common tests should be described solely by name; describe more complex techniques in the Methods section.*
☐	☒	A description of all covariates tested
☐	☒	A description of any assumptions or corrections, such as tests of normality and adjustment for multiple comparisons
☐	☒	A full description of the statistical parameters including central tendency (e.g. means) or other basic estimates (e.g. regression coefficient) AND variation (e.g. standard deviation) or associated estimates of uncertainty (e.g. confidence intervals)
☐	☒	For null hypothesis testing, the test statistic (e.g. *F*, *t*, *r*) with confidence intervals, effect sizes, degrees of freedom and *P* value noted<br>*Give P values as exact values whenever suitable.*
☐	☒	For Bayesian analysis, information on the choice of priors and Markov chain Monte Carlo settings
☐	☒	For hierarchical and complex designs, identification of the appropriate level for tests and full reporting of outcomes
☐	☒	Estimates of effect sizes (e.g. Cohen's *d*, Pearson's *r*), indicating how they were calculated

Our web collection on statistics for biologists contains articles on many of the points above.

Software and code

Policy information about availability of computer code

Data collection	Breath-by-breath data were collected using the MetaMax 3B-R2 system. Smart shirt sensors were collected using the Hexoskin iOS app and HxServices/HxConvert software.
Data analysis	Custom deep learning code using TensorFlow libraries. Signals were analyzed in Matlab R2020b. Statistics were conducted in R (v3.5.1).

For manuscripts utilizing custom algorithms or software that are central to the research but not yet described in published literature, software must be made available to editors and reviewers. We strongly encourage code deposition in a community repository (e.g. GitHub). See the Nature Research guidelines for submitting code & software for further information.

Data

Policy information about availability of data

All manuscripts must include a data availability statement. This statement should provide the following information, where applicable:

- Accession codes, unique identifiers, or web links for publicly available datasets
- A list of figures that have associated raw data
- A description of any restrictions on data availability

Data are available by contacting the corresponding author upon reasonable request.

Field-specific reporting

Please select the one below that is the best fit for your research. If you are not sure, read the appropriate sections before making your selection.

☒ Life sciences ☐ Behavioural & social sciences ☐ Ecological, evolutionary & environmental sciences

For a reference copy of the document with all sections, see nature.com/documents/nr-reporting-summary-flat.pdf

Life sciences study design

All studies must disclose on these points even when the disclosure is negative.

Sample size	The sample size of n = 22 for development of a new proof-of-concept V̇O2p predictor is similar to previously published works aimed at estimating V̇O2p from wearable sensors, such as Beltrame et al. (Sci Rep. 2017;7:45738)(n = 16), and Altini et al. (IEEE J Biomed Heal Informatics. 2016;20(2):469–75)(n = 22)
Data exclusions	No data were excluded
Replication	Results were confirmed by both primary authors.
Randomization	Participants' whole data were split into either train, test and validation sets using random permutations using computer code and split according to the pre-set percentages.
Blinding	Participant allocation was performed after collection, so researchers were inherently blinded to group allocation during collection. As stated above, groups were performed via randomization. Analysis was performed objectively using standard accuracy metrics, and was thus not privy to possible observer bias.

Reporting for specific materials, systems and methods

We require information from authors about some types of materials, experimental systems and methods used in many studies. Here, indicate whether each material, system or method listed is relevant to your study. If you are not sure if a list item applies to your research, read the appropriate section before selecting a response.

Materials & experimental systems

n/a	Involved in the study	
☒	☐	Antibodies
☒	☐	Eukaryotic cell lines
☒	☐	Palaeontology and archaeology
☒	☐	Animals and other organisms
☐	☒	Human research participants
☒	☐	Clinical data
☒	☐	Dual use research of concern

Methods

n/a	Involved in the study	
☒	☐	ChIP-seq
☒	☐	Flow cytometry
☒	☐	MRI-based neuroimaging

Human research participants

Policy information about studies involving human research participants

Population characteristics	Twenty-two young healthy adults (13 males, 9 females; age: 26±5 yr; height: 1.71±0.08 m; mass: 70±11 kg; peak oxygen uptake: 42±6 ml/min/kg) with no known musculoskeletal, respiratory, cardiovascular, or metabolic conditions.
Recruitment	Participants were a convenience sample from the area near the University of Waterloo. Posters and word-of-mouth were the methods used to recruit participants. Since this was a convenience sample, it is possible that participants who volunteered to participate in the study may not be completely representative of the population of young healthy adults.
Ethics oversight	The study was approved by a University of Waterloo Research Ethics committee (ORE #32164) and conducted in accordance with the Declaration of Helsinki

Note that full information on the approval of the study protocol must also be provided in the manuscript.
